# Stakeholder perspectives of immunisation delivery for adolescents with disability in specialist schools in Victoria, Australia: ‘we need a vaccination pathway’

**DOI:** 10.1186/s12889-024-19322-y

**Published:** 2024-07-23

**Authors:** Jane Tuckerman, Yasmin Mohamed, Frances Justice, Tove Andersson, Kerryann Wyatt, Kate Broun, Alice Bastable, Isabella Overmars, Jessica Kaufman, Margie Danchin

**Affiliations:** 1 Faculty of Medicine, Dentistry and Health Sciences, University of Melbourne, 161 Barry St, Carlton, VIC 3010 Australia; 2 Murdoch Children’s Research Institute, Royal Children’s Hospital, 50 Flemington Road, Parkville, 3052 Australia; 3 Centre for Behavioural Research in Cancer, Behavioural Science Division, Cancer Council Victoria, 615 St Kilda Rd, Melbourne, VIC 3004 Australia; 4 Prevention Division, Cancer Council Victoria, 615 St Kilda Rd, Melbourne, VIC 3004 Australia; 5 Royal Children’s Hospital, 50 Flemington Road, Parkville, 3052 Australia

**Keywords:** Disability, Adolescent, Vaccination, Special school, HPV

## Abstract

**Background:**

Adolescents with disability have lower vaccination rates than the general population, including HPV vaccination. Understanding the multi-level influences on vaccination in specialist schools is crucial to achieve optimal vaccination coverage and vaccination experiences for adolescents living with disability.

**Objective:**

To identify and improve understanding of the facilitators and barriers of HPV vaccination among adolescents with intellectual disabilities or autism in Victorian specialist schools to inform strategies to increase vaccination acceptance and uptake.

**Methods:**

Qualitative interviews with key stakeholders (adolescents with disabilities, parents, school and council immunisation staff) from six specialist schools in Victoria, Australia. Data were analysed thematically. Inductively derived themes were then deductively mapped across the UNICEF ‘Journey to Immunization’ model.

**Results:**

32 interviews were conducted with stakeholders (2 adolescents, 7 parents, 13 school staff, 10 council staff). Trust in vaccines was high, but knowledge of the HPV vaccine was limited. Barriers included lack of accessible information for parents, the consent process, behavioural challenges and vaccine-related anxiety among students. The immunisation program in special schools was perceived as convenient, however preparing students for vaccination day and catering to individual student needs were key. Participants expressed a need for more parent information about options and additional support for vaccination outside of the school program.

**Conclusions:**

Our study identified a range of facilitators and barriers to the school immunisation program for students with disabilities in specialist schools. The next phase of this work will use co-design workshops to build on the suggestions for improvement and opportunities that could be leveraged to improve vaccination uptake.

**Supplementary Information:**

The online version contains supplementary material available at 10.1186/s12889-024-19322-y.

## Introduction

People with disability are more vulnerable to complications from vaccine-preventable diseases, frequently face additional barriers to accessing healthcare and are at risk of under or non-immunisation [1–3]. A recent global focus highlighted the importance prioritising vaccination for GEDSI (Gender Equality, Disability and Social Inclusion) populations and improving vaccine equity in populations with disability.

In Australia, the National Immunisation Program (NIP) funds adolescent vaccines, including human papillomavirus (HPV), diphtheria-tetanus-pertussis (dTPa) and Meningococcal ACWY. The HPV schedule has evolved: [4] as of 2023, a single dose nine valent HPV vaccine is given in year 7 (12–13 years old) [5]. Previously, it was two doses six months apart, and before 2018, three doses [4]. The HPV vaccine is particularly important for people with disability, who may face unique barriers to cervical screening, diagnostic testing and access to cancer treatment later in life [6].

However, adolescents and young people with disability have lower rates of vaccination than the general population [2]. A previous Australian study found HPV vaccine coverage in adolescents with developmental disabilities was below national levels [7]. In another study, special education schools were 5.6 times more likely to have low coverage of the first dose of HPV vaccine [8]. More recently, a 2017 study in Victorian specialist schools found that only 41% of students received all three recommended HPV doses, compared with 75% of their mainstream counterparts, with a similar discrepancy for the dTPa vaccine (63% versus 89%).^9^

In Australia, vaccines for adolescents funded through the National Immunisation Program (NIP) include human papillomavirus (HPV), diphtheria-tetanus-pertussis (dTPa) and Meningococcal ACWY. In Victoria, these vaccines are delivered at school through the Secondary School Immunisation Program. An estimated 380,000 school students (aged 5–18) in Australia have some level of disability, with approximately 12% of adolescents with a disability attending specialist schools [9]. There is significant heterogeneity between student disability types [9] and specialist schools are classified into five types; four cater for intellectual disability (special school, special development school, multi-mode school) or autism, while the fifth caters for children with significant physical disability.

There are a lack of adequate professional guidelines to support immunisation delivery in special schools including how to hold students with disabilities and deliver immunisations effectively and safely, in particular for adolescents with intellectual disability and autism [10].

To optimise coverage and vaccination experiences for adolescents with disability it is important to understand the factors that influence HPV vaccine uptake in this population when delivered as part of a school-based program. Equally, an accurate understanding of barriers to HPV vaccination can facilitate development of tailored interventions for adolescents with disability through stakeholder engagement and collaboration.

The aim of the Developing Optimised Vaccination Engagement in Specialist Schools for Human Papillomavirus (DOVES [HPV]) project was to improve understanding of the facilitators and barriers to HPV vaccination among adolescents with intellectual disabilities or autism in Victorian specialist schools to inform strategies to increase HPV vaccination acceptance and uptake. DOVES was a three-phase qualitative study with a focus on HPV. Phase I included a literature review, Phase II interviews with key stakeholders and Phase III consisted of co-design workshops to develop strategies to improve vaccination; here we report phase two, which aimed to identify barriers and enablers of vaccination among adolescents with disability in specialist schools.

## Methods

### Design

Semi-structured interviews were conducted with stakeholders at participating specialist schools. The decision to employ qualitative interviews exclusively was based on the recognition that depth of understanding, nuanced insights, and the ability to explore underlying motivations and perceptions in detail were paramount in exploring the complex and multifaceted barriers faced by parents and adolescents with disability regarding vaccination. While a survey may have reached a wider audience, qualitative interviews captured the richness and subtleties of individual experiences, beliefs, and concerns, thus providing a more comprehensive understanding necessary for targeted intervention strategies. This qualitative study is presented using the Consolidated Criteria for Reporting Qualitative (COREQ) research [11].

### Participants and recruitment

Schools were recruited first, followed by stakeholders associated with each school. Four specialist school types were eligible: special, special development, multi-mode and autism specific. We excluded physical disability schools due to the unique differences this cohort faces for vaccination, such as being unable to move independently, and therefore having different barriers to immunisation. The study team obtained a list of eligible specialist schools in Victoria and contacted the school principal by phone or email to invite the school to participate. We purposefully selected a mix of metropolitan and regional specialist schools. Written consent was obtained from the principal who facilitated identification and recruitment of potential participants within the school community including adolescents, parents and school staff (including immunisation coordinators, school principals, and teachers). Invitations were sent by the school on behalf of the research team or contact details were provided to the research team. Participants could also contact the research team directly.

Council immunisation providers (including nurses and program managers) associated with each participating school were also contacted to participate in the study. Written consent was obtained from all participants. For adolescents, parental consent and assent from the adolescent was obtained. A research team member contacted participants by email or phone to schedule interview times. Participants were compensated for their time with a gift voucher. Ethics approval was received from the Royal Children’s Hospital Human Research Ethics Committee (HREC/79,238) and the Department of Education and Training, Victoria (2021_004517).

### Data collection

Two authors (JT; female; PhD Paediatric Medicine and YM; female; MPH) undertook the majority of semi-structured interviews, while a further two researchers (JK; female, PhD Public Health and IO; female; MPH) completed some interviews. All interviews were conducted individually with participants, except in the case of adolescents with disabilities who were invited to have a parent or another person present. The project team developed the interview guide (Supplementary material) from which all interviewers worked from; any revisions were discussed at team meetings. Interviews were conducted between June and September 2022 using Zoom [12], audio recorded and transcribed verbatim by a transcription service (Outscribe^®^) [13].

### Data analysis

**Fig. 1:**
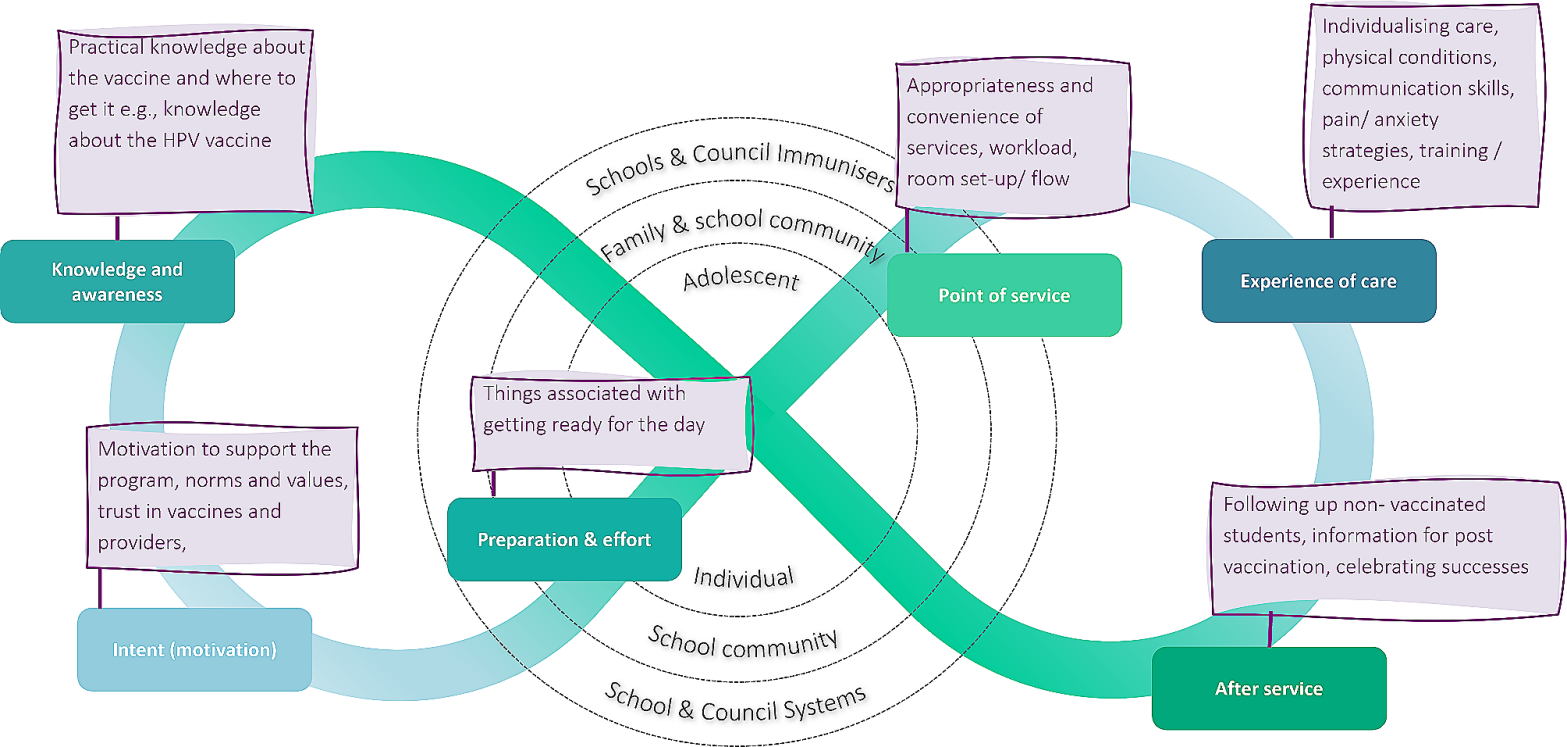
Journey to Immunisation (adapted from UNICEF) [17]

The two primary researchers (JT and YM) coded the qualitative interviews inductively using a thematic approach [14], aided by NVivo [15]. To ensure consistency, the first three interview transcripts were analysed independently by two researchers (JT and YM). Coding was discussed, and a framework agreed upon before additional interviews were analysed by both researchers. Regular meetings were convened with the research team to discuss themes. The research team used the UNICEF ‘Journey to Immunization’ model to present the key findings from the interviews [16, 17]. This model (Fig. 1) uses a human-centred design approach to consider each stage of the vaccination experience: before, during and after the point of vaccination. The themes were identified inductively (Table 1) to explore participants’ experiences and perspectives in more depth. These themes were then mapped deductively to the categories in the UNICEF ‘Journey to Immunization’ model as we felt this framework best represented all barriers encountered and that need to be addressed to develop a new immunisation pathway for this priority population.

**Table 1:** Barriers and facilitators

Knowledge and awareness	Intent (motivation)	Preparation & effort	Point of service	Experience of care	After service
**What is working…**					
• Most parents support vaccination	• Most schools and councils highly motivated to support the delivery of vaccines in SS • Vaccination seen as a social norm • High levels of trust in vaccines and government / health care providers • Few differences in attitudes between HPV and other vaccines	• Most schools prepare adolescents for the day – preparation seen as key to success • Most schools-councils work well and have established on-going relationships	• School program enables many adolescents (& parents) to access vaccinations (convenient and overcome disability challenges) • School is a safe place for many – need to protect this	• Council and school staff work hard to support the individual needs of students on the day • Additional (annual) vaccines normalise vaccines • Most adolescents supportive of each other • Most adolescents OK (and proud) once vaccine given	• Councils attempt to catch-up students at follow-up vaccination days at the school • Councils support delivery in other settings (council clinic) or refer on to clinics with sedation available (hospitals)
**Barriers**					
• Difficult to gauge level of knowledge among parents (information sent out to parents but no follow-up) • Information overload for parents • Resources not accessible to all parents (CALD families, low health literacy) • Lack of understanding of importance of HPV vaccine – not needed for boys or adolescents with a disability • HPV vaccine is just for cervical cancer • Adolescents associate vaccines with pain, discomfort, anxiety (limited awareness of benefits)	• People making decisions about the facilities may not be aware of specific needs • Those responsible for providing consent forms/ responding to questions may not see immunisation as a priority • Parents may not feel they are able to attend immunisation session at school • Lack of knowledge among parents about the process	• Uncertainty if paper-based consent forms sent home with children reach parents • Parents may not understand consent form / process • Challenges with following up consent forms – lack of resources and inconsistencies • Councils not always aware of the specific needs of each adolescent • Limited avenues for parents’ questions to be answered and school staff not always equipped to respond • School staff engagement varies between schools	• School seen as a safe place – don’t want to jeopardise • Parents’ understanding of limitations of staff to administer vaccines • Attendance challenges • Limitations of existing facilities / spaces available (small rooms, lack of flow) • HPV vaccine ‘stings’	• How to best manage student anxiety and behavioural challenges within limitations • Challenges with catering for individual student needs (parents attending, room space, preparation) • Level of comfort with disability (council) and assisting with vaccines (school staff)	• Lack of process to follow up vaccination day fails • Lack of process to follow up vaccination day absences • Parents do not always know how they can access sedation • Follow-up settings (other clinics/ GP) not always appropriate and may burden parents. • Parents may be less inclined to follow up HPV rather than have opportunities to get DTP ( e.g. injury)

Footnote: CALD: Culturally and linguistically diverse

## Results

Six Victorian specialist schools agreed to participate and 32 school and council staff, parents, and adolescents participated in interviews (Table 2). Although the intention was to recruit schools only if the school principal, immunisation coordinator, and respective council providers consented to participate, one school was included that contributed school staff only and another contributed only school and council staff. The adolescent interviews lasted up to six minutes, with all other interviews lasting between 15 and 50 min.

**Table 2:** Participant characteristics

Participant group	Principal / Vice Principal	Other school staff*	Council staff	Parent	Adolescent
	*N* = 6	*N* = 7	*N* = 10	*N* = 7	*N* = 2
School					
1 - Urban	1	2			
2 - Urban	1	1	3		
3 - Regional		2	2	2	2
4 - Regional	1	1	2		
5 - Regional	1	1	1	5	
6 - Urban	2		2		
Gender					
Female	4	6	10	6	1
Male	2	1		1	1

Footnote: * Includes one immunisation coordinator, one school nurse, one support staff, and four teachers

### Interview findings

The key themes associated with each of the stages in the Journey to Health and Immunization framework are presented below, with barriers and facilitators summarised in Table 1. Participant suggestions for future program improvements are presented in Table 3, which will be taken forward to the third phase of this DOVES HPV project. While we selected schools to represent both urban and regional areas, we observed few differences in findings between these geographical regions. One notable difference, however, was in access to follow-up services in the event of an unsuccessful vaccination day and this is elaborated below.

**Table 3:** Interventions suggested by interview participants

Category	Suggestion
Information, education, and communication for parents	Have information and consent forms available in other languages
	More effective ways to communicate to parents with culturally and linguistically diverse backgrounds
	Simplify information for parents – use more visuals
	Better communication between school and parents & formats
	Educating parents on the importance of vaccination
	Establish resources for schools and teachers Written information to put in school communication chains e.g., newsletters or printed off and handed to parents at pick-up.
	Information session for parents
	More information on the process to help parents prepare adolescents.
	Information specific to people with disability
	Adapt information that goes out to parents to specialist schools Reassurance to parents that vaccination will occur in a safe space
Information for school staff	Having schools explain the vaccination process in a basic level – a rundown for staff just to have the knowledge to be able to pass onto their students.
	Overall promotion of the school vaccination program to parents/school staff
Individual student plan	School staff and councils to work with parents to develop a tailored approach for each child
	Document a clear plan for each student (parents and school staff)
	Strategies for adolescents with behavioural challenges
Preparation for students	Teachers preparing students
	Age-appropriate education package about what is going to happen
	Pre-warning- need visuals and simple concise instructions, to explain why we need our vaccines.
Setting / space set up	Provide a setting that adolescents can see their peers, go together, have distraction, sit down afterwards.
	Vaccination day set up that allows flow and not pre/post cross over
	Individualise space – it needs to feel like a safe space for students
	The right staff are put on when there is an immunisation
Distraction techniques	Use distractions – iPad, phone, TV
	An education [support person] to support the students through immunisation.
Parents given the opportunity to attend	Communicating the date of vaccination day with parents, giving parents the opportunity to attend

### Knowledge & awareness

Parents, staff and adolescents had limited knowledge about HPV, vaccines and the vaccination day process: *“ …I think HPV is transmissible, I think”* (ID# 68, Parent), *“I don’t know what it involves and what’s in it.”* (ID# 40, Immunisation coordinator) and *“if you asked me to tell you what HPV [the vaccine] does, like, what it stops, I would struggle to tell you.”* (ID# 62, Assistant Principal). Neither of the two adolescent participants articulated the benefits or reasons for vaccinations, instead describing feeling “scared” on vaccination day and “terrified” of the needles. School staff reported lower levels of knowledge among parents from culturally and linguistically diverse (CALD) backgrounds and those with low levels of literacy and/or intellectual disability in particular. Many parents spoke about being overloaded with vaccine information and there was an expressed need to provide more accessible information to parents, that enabled everyone the “*opportunity to understand*”. (ID# 39, School staff)

Knowledge and experience with disability was mixed among immunisation staff. Many council immunisers did not have specific disability training, with some perceiving the specialist school environment could be challenging, particularly for staff with limited disability experience. Councils tried to send the same nurses to specialist schools each year to ensure a continuity of skills and experience: *“…we’ve had the same nurses come in and they tend to not only know us and recognise us, they know the kids too.”* (ID# 73, Principal).

### Intention & motivation

Parents expressed high levels of trust in vaccines and vaccine providers, and vaccination in general was seen as a social norm and necessary to protect people at the population level, *“…it’s part of what we do as a society and the community to keep everyone safe…”* (ID# 59, Parent). Parents did not express specific beliefs about the HPV vaccine and most council and school staff perceived no difference in parental attitudes between HPV and other vaccines given during adolescence, reporting that parents either consented to all vaccines or none. However, some council nurses recalled instances where parents had not consented based on the child’s level of disability.

*“I had a mum […] she said I’m only consenting to Boostrix, [name] is severely disabled, and I don’t believe she’s ever going to have sex, so I don’t think there’s a need for her to have the HPV vaccine.”* (ID# 41, Council).

Some non-consenting parents were reluctant to send their children to school on vaccination day, and returned incomplete consent cards for fear they would be vaccinated against their wishes, “I know one parent, if they know it’s vaccination [day], … she doesn’t send her child here because she doesn’t want them [vaccinated]”. (ID# 40, School immunisation coordinator) Most school and council staff saw vaccines as important for the health of adolescents with disability and had positive attitudes toward the school immunisation program, believing it was convenient for parents. In general, school staff were motivated to support the school immunisation program, though some were more engaged than others.

### Preparation & effort

HPV vaccine information was disseminated to parents by schools on behalf of the councils. Council staff rarely had the opportunity to speak with parents directly, making it difficult to gauge their level of knowledge. While the parents interviewed felt they had access to enough information about vaccines, many wanted greater detail about vaccination day. Providing accessible information to parents was seen to be particularly challenging for those with low literacy or having intellectual disabilities themselves.

*“… I’d say quite a big percentage of [parents] would be running their lives on a day-to-day basis. Because a lot of our families have IDs [intellectual disabilities] themselves, realistically… so there’s some families with paperwork, it’s too daunting for them.”* (ID# 72, Assistant Principal).

Many aspects of the consent process were challenging. Council staff relied on a list of dates of birth rather than a pre-existing classroom list to determine eligibility, making it challenging to track and identify eligible adolescents. Both school and council staff highlighted the ambiguity over responsibility for contacting parents to follow up on consent forms, with much reliance placed on parents to return the consent card. While the consent process worked well in some schools, it tended to rely on dedicated individuals rather than clearly defined procedures.

School staff and parents talked about the importance of knowing the date and the process of vaccination day so that they had adequate time to prepare the adolescents, and for parents to raise any concerns with teachers. Social stories or stories which describe a particular situation, event or activity were seen as a useful way to prepare the adolescents and *“explain what’s happening on the day*” (ID# 44, School staff). However, one school staff member felt that in some cases it was better not to prepare adolescents in detail for vaccination day, perceiving this, “*just heightens anxiety because it happens so infrequently”* (ID# 74, Assistant Principal). Many participants talked about a need for individual plans to prepare the adolescents and tailor approaches, because “*it’s different for every student.”* (ID# 73, Principal).

Most of the schools and councils reported working together to plan vaccination day, though the level of school to council engagement varied, as did the school staff responsible for liaising with council. Participants felt the relationship between council and school staff was important, but some felt that the coordination could be improved.

### Point of service

The school immunisation program was seen as enabling accessible vaccination for families “*who would struggle to get it done otherwise”.* (ID# 62, Assistant Principal) Parents agreed that the service was convenient, though one parent said the real challenge was *“relinquishing control in not being there”* with their child (ID# 66, Parent). Parents placed their trust in the school, which they and the school and council staff all perceived as a “safe place” for adolescents with disability. They stressed the importance of a familiar environment, routine, and staff, with one parent highlighting how much easier it was to vaccinate her child at school compared to at a GP or other unfamiliar and unsupportive environment.

Participants highlighted several challenges to vaccination day logistics, like the order of vaccines and the limited-service delivery space. Most schools had a systematic process to follow up absent students, though some council staff also reached out to parents directly. Inevitably, some adolescents missed out completely on vaccines if they were not captured in the school program.

### Experience of care

Multiple participants highlighted the importance of the right physical environment on vaccination day. This included the room size and set up, and the availability of things to distract or reward the adolescents such as iPads, music, lollies, or bubbles. For one adolescent, the most important thing was to be distracted, *“I don’t look at it [the needle].”* (ID# 47, Student) While many thought that it was beneficial for adolescents with disability to be able to see their peers, others felt it was better to keep the students in smaller groups because, “*you don’t want to put other students at risk if another student’s going to escalate too much.”* (ID# 39, School staff).

Council and school staff said it was common for adolescents with disability to have anxiety about the vaccines, and one adolescent described feeling scared during their vaccination. Adolescents with disability were deemed to be more perceptive to their environment and despite much preparation it could all backfire quickly, particularly if confronted with anything unfamiliar or inexperienced people. Both council and school staff talked about abandoning vaccination if it became too difficult.

School staff described their reluctance to restrain students and given the strength of some adolescents, it was not always safe to attempt vaccination. Staff did their best to “*really try and avoid, obviously, physical restraint*” (ID# 58, Council) however this added its own challenges with limitations around the use of restraint. School parents often had an expectation for the school to achieve vaccination and to just, ‘hang onto them for a minute’ or ‘grab’ them ‘until it’s done’, conflicting with school staff who feared ruining the teacher-student relationship and did not know how to support an adolescent in that situation. While parents sometimes accompanied their child on vaccination day to help with this process, one school staff participant questioned the longer-term impact.

*“…one of my students that has to be pinned down; I’m not restraining anyone, so the family does have to [do it], but is that the best for that individual long-term, or is that causing trauma around vaccinations? Probably.*” (ID# 39, School staff).

School and council staff felt that the vaccination day experience for most students was positive and described how proud some students were once vaccinated. One participant believed that adolescents with disability were less anxious and less distressed about vaccines compared with students in mainstream schools, attributing this to them being, “*used to seeing different levels of anxiety and stress and behaviours in their day-to-day classroom.”* (ID# 75, Council).

### After service

While very few students could not be vaccinated at school, when the vaccinations were unsuccessful it was often disappointing for school and council staff, with many reporting that the most difficult part was, “*not meeting the expectation of the parents”* (ID# 62, Assistant Principal). Unsuccessful vaccination also placed an additional burden on parents and extra effort was then required to ensure successful vaccination elsewhere, *“[…] we had to take him to the GP which took us six months of training at home.”* (ID# 45, Parent).

When student vaccination was unsuccessful, council or school staff would notify the parents and provide information about alternative options. However, the onus was on parents to navigate these services. Apart from the initial contact with parents to state that their child could not be vaccinated and to provide information about alternative services, there was no clear process for follow up or support. Some parents wanted comprehensive information in advance about the various alternative options available, which schools were not able to provide. This issue was particularly felt by regional parents, who had more limited access to follow-up services. Urban areas generally had better access to these tertiary services and some information on where to locate them compared to regional areas, although this information was still inadequate.

## Discussion

Lower rates of vaccination for adolescents with disability represent an unacceptable health inequity. To our knowledge, this is the first study to explore the influences on participation in and experiences of immunisation in specialist schools from the perspective of adolescents with disability, their parents, council immunisation staff and school staff. Our qualitative interviews highlighted a range of diverse experiences occurring before, during and after immunisation service delivery in specialist schools. The main drivers of vaccination for adolescents with disability, including HPV vaccines, were having accessible parent information. As well as this, addressing structural factors for care that systematically incorporates consent, adequate preparation and/or management of adolescents for vaccination day and a clear vaccination pathway.

Although HPV vaccine coverage is lower in adolescents with disability [10], meaning that eliminating cervical cancer for this population will take longer, we did not identify a difference in attitudes between HPV and other routine vaccines. Parents in our study were all favorable to the HPV vaccine and did not question the need for HPV vaccination in adolescents with disability. Studies suggest that parents may believe that the HPV vaccine is ‘not needed’ for their adolescent with disability – potentially due to a lack of recognition of the importance of appropriate sexual health education in this population [18, 19]. In other studies, parents of adolescents with disability who did not consent to HPV vaccination have cited reasons like “not needed”, “not sexually active” [18, 20] and “children with disabilities to be less likely to become infected with HPV” [21]. While the parents we interviewed did not share this belief, some immunisation providers did raise concerns about parents not perceiving the HPV vaccine as necessary for their adolescent with disability, suggesting this may be an area for further exploration and education.

The logistical barriers identified in our study are similar to other research from the US and Australia [10, 22], however there are limited studies in this area. Likewise, the current services and resources available to support the vaccination of students with disabilities mirror those provided to mainstream students. While, in principle, these students can access additional resources, they are subject to the same consent, preparation, supports and referral processes as mainstream students, revealing an evident unmet need [2]. One US study identified a need for tailored information about the school immunisation program, environmental considerations, to have healthcare personnel familiar to the students and explicit, ethically stringent policies on the use of physical restraint [22]. Like our study, previous Australian research identified challenges with consent, high levels of school absenteeism, student anxiety, and a lack of professional guidelines around delivering immunisations effectively and safely to adolescents with intellectual disability [10]. Our findings extend this previous work by identifying the extent to which structural processes influence parents - and in turn students - engagement in the program. The fact that the school immunisation program operates with schools receiving no financial reimbursement, may explain the variation and lack of consistency between schools and councils’ approach.

When considering costs, it is important to include reimbursement of adequate resource allocation for schools to support logical barriers, staffing costs, infrastructure and equipment, communication and outreach, data management and reporting as well as collaboration and coordination. By addressing these funding-related challenges in the context of school immunization programs, stakeholders could work towards improving consistency, effectiveness, and sustainability in immunization efforts within educational settings.

While there are limited resources on vaccinating adolescents with disabilities or resources available for schools, they lack the detail that parents need and do not provide detail of referral pathways, if required, beyond the school program. The limited accessible parent information that caters to CALD parents or parents with low literacy is an important finding and further illustrates the need, beyond suitable resources, for well-planned structural supports and systems to support all phases of the program. There is a paucity of research examining interventions that specifically target vaccination for adolescents with disability, although an individualised approach is considered preferable [23–25]. As our participants highlight, there are a multitude of things that could be considered, all of which will be taken forward to the third phase of this DOVES HPV project.

Our finding regarding the lack of referral pathways in cases of unsuccessful vaccination, with the onus on parents to navigate services is important and highlights a critical health system gap. This gap indicates a systemic issue where parents, already burdened by the complexities of healthcare navigation, face additional challenges without adequate support. The preference for school-based vaccination programs, instead of in general practice, suggests a need for more accessible and structured vaccination pathways within the education system, which could alleviate the pressure on parents and ensure higher vaccination uptake. Parents need greater awareness of tertiary-based services that offer both distraction and awake sedation for children with unsuccessful school-based vaccination, and how to access them. Addressing these gaps could lead to more tailored vaccination that meet the needs of parents and adolescents, thereby improving overall vaccination rates and reducing barriers to access.

Our work highlights the need for a consistent, structured pathway for this population to receive HPV vaccines safely and ethically, and these findings are also relevant for other vaccines delivered through the school immunisation program. The participants in our study recommended and emphasised the need for the development of several key strategies, such as improved preparation for the day, accessible parent information and practical solutions such as individual student plans, setting up the right vaccination environment and a clear vaccination pathway. Moving forward it will be important to consider the need to co-design future interventions to improve vaccine uptake with the community, including adolescents with disability, looking at individual barriers as well as any systems change required. This could be achieved using the principles of human centred design or action-based research. Suggestions from participants are an important starting point from which to further develop interventions to improve vaccine uptake among adolescents with disability.

### Limitations

There are some limitations to this study. While we sampled the range of stakeholders, providing a diverse range of perspectives, this approach may have reduced opportunities for saturation. This study was undertaken in 2022 and the recruitment of schools and the ability to engage parents and adolescents was likely impacted with communities exhausted from two years of the COVID-19 pandemic. Only two adolescents were included in the study, which was lower than intended and their perspective is limited. Recruiting adolescents presented significant challenges, such as securing the interest and availability of the parent or carer and the adolescents. While we partnered with schools to facilitate access, future research may benefit from strategies such utilising digital recruitment methods like social media to reach adolescents more effectively, and involving adolescents in the study design to ensure the approach resonates with their interests and needs. Furthermore, reassuring adolescents that they will be fully supported during the research process and can participate with their peers and parents, may increase willingness to participate. The participating parents may not be representative of other parents at their adolescents’ school or parents of an adolescent with a disability more generally. Notably our sample did not include parents directly opposed to the HPV vaccine. The perspective of these parents is important to consider, particularly when designing new strategies to improve uptake.

## Conclusion

Adolescents with disability in specialist schools face unique challenges to engage with school-based immunisation programs. We identified a range of facilitators and barriers to the current Victorian school immunisation program, as well as suggestions for improvement. Future phases of work will seek to co-design solutions and develop a pathway for the delivery of vaccines in the school immunisation program. This approach will build on the current and previous work and provide feedback and input to the next phases of the project.

### Electronic supplementary material

Below is the link to the electronic supplementary material.

Supplementary Material 1

## Data Availability

The datasets generated and/or analysed during the current study are not publicly available but are available from the corresponding author on reasonable request.
