# Supplementary material for: Stakeholder perspectives of immunisation delivery for adolescents with disability in specialist schools in Victoria, Australia: ‘we need a vaccination pathway’
Source: BMC Public Health. 2024 Jul 23;24:1973. doi: 10.1186/s12889-024-19322-y (PMC11267833; doi:10.1186/s12889-024-19322-y)
Supplement: Supplementary file 1 — Supplementary Material 1 [file 12889_2024_19322_MOESM1_ESM.pdf]

79238 Phase I – Semi-structured interview guide (Parents/Guardians)

Explain the study – We are aiming to better understand the needs of adolescents with disability receiving vaccines at school and how we might improve this process.

Before we get started, I just want to highlight some things about the interview.

- You can stop participating at any time, or ask to skip a question if you do not wish to answer it
- Your interview today will be recorded on secure software and then typed up.
- Once we complete the interview we will remove your name from the data we collect and when we write the study report you will be identified by a participant number.
- The interview will last about 45 minutes
- You will receive a gift card at the end

Questions?

Check - Consent Form / PICF

Demographic questions

Interviewer note please read each question separately.

First, I am going to ask you some questions about yourself.

1. How old are you?
2. What is your gender?
3. What languages do you normally speak at home?
4. How many children do you have? How old are they?
5. Who lives in your household with you?
6. What kind of work did/do you do? Do you work full-time/ part-time? Paid or volunteering?
7. (Ask if not already known) Do you live in a metropolitan, or rural, or remote area?

8. **Could you tell me something about yourself, and your child's condition?** Probe: discuss identities and life experiences however they best resonate with parent, child's disability, wider family, etc.)

Domain 1: General knowledge and attitudes of school vaccination programs

I am going to ask you a few questions about vaccination and school vaccination programs.

9. **What are your thoughts on school vaccination programs in general?**

   Probe: What do you like or not like about them? Why? What worked well? What needs to improve?
   Probe: Do you prefer your child having vaccination at school or a GP? Having it with or without your presence? Why?
   Probe: Are there any stories or experiences (from you or other people you know) that you could share with us about school-based vaccination of adolescent with disabilities?

10. **Can you walk me through how you make, or have made, decisions about vaccinations for your child/ren?**

    Probe: Was there things that made it hard? What helped?
    Probe: Do you discuss your views on vaccination with your child/ren?

Domain 2: Knowledge and attitudes of specific vaccines

I am going to ask you a few questions about the vaccines more specifically.

11. **Tell me about how you [or other parents/guardians] usually get information about vaccines for your adolescent ?**

    Probe: Who would you talk to if you had questions or concerns?
    Probe: What resources have you accessed?
    Probe: Anything that has been particularly helpful?
    Probe: Where/how/from whom would you prefer to get this information?
    Probe: Is there any information about vaccinations for your child that you would like to know but haven't been able to find out about?

12. **What do you know about HPV vaccination?**

Probe: Purpose, safety, number of doses, administration, side effects, etc.?)
Probe: Would you (or have you) give consent for your child to receive the vaccine if it is available in schools? Why would/would not?
Probe: Do you discuss HPV vaccination with your child/ren?
Probe: Do you discuss your views on HPV vaccination with your child/ren?
Probe: Do you think your child is at risk of HPV ? Why / why not?
Probe: Have any issues or disagreements come up in discussing HPV vaccination with your family or community?
Probe: Are there circumstances in your life or your child's life that you think affects your thoughts about, or access to, HPV vaccination?

13. **What about the other vaccines given to secondary school students…**

Probe: e.g., dTpa (the diphtheria-tetanus-whooping cough vaccine) and MenACWY (the meningococcal vaccine)?

14. **What do you know about COVID-19 vaccination?**

Probe: Purpose safety, number of doses, administration, side effects).
Probe: Would you give consent for your child to receive the vaccine if it is available in schools? Why would/would not?

Domain 3: Vaccination day

I am going to ask you a few questions about vaccination day.

15. **Has your child with a disability received any vaccinations at school?**

Probe: Which ones?
Probe: Do you have other children who have received vaccinations at school?
Probe: If the interviewee has a child without disability, explore if there are any differences in, and the reason behind, vaccinating the children with and without disability.)

16. **What role has your child's school played in your decision-making consenting to receiving a vaccination for your child?**

    Probe: Communication from the school? Role of teachers? Role of nurses? Other staff?

17. **Can you tell me about how vaccination day worked [or didn't work] for your child?**

18. **Can you tell me how you think [child's name] felt about vaccination day?**

19. **Can you tell me a little bit about how you were prepared for vaccination day?**

20. **Can you tell me a little bit about how [child's name] was prepared for vaccination day?**

Domain 4: Possible interventions

21. **If we want to encourage more students with disability to be vaccinated and to make this a more positive experience for everyone, what do you think we should do?**

    Probe: What would be helpful for young people with disability?
    Probe: How can we help minimise anxiety, pain or distress?
    Probe: What kind of information or support do parents/guardians need?
    Probe: What do you think is the most helpful ?strategy ?resource?
    Probe: What are the roles for schools, governments, or others in supporting vaccination?

22. What would you recommend as more effective ways to communicate information about different types of adolescent vaccines to parents?
Probe: Face-to-face workshops, pamphlets, messaging**?**

<u>Wrap up</u>

23. Regarding vaccination or school vaccination programs in general, is there anything else you would like to share with me?
